# Staff perspectives on the influence of patient characteristics on alarm management in the intensive care unit: a cross-sectional survey study

**DOI:** 10.1186/s12913-023-09688-x

**Published:** 2023-07-05

**Authors:** Felix Balzer, Louis Agha-Mir-Salim, Nicole Ziemert, Malte Schmieding, Lina Mosch, Mona Prendke, Maximilian Markus Wunderlich, Belinda Memmert, Claudia Spies, Akira-Sebastian Poncette

**Affiliations:** 1 Institute of Medical Informatics, Charité – Universitätsmedizin Berlin, Corporate Member of Freie Universität Berlin and Humboldt-Universität zu Berlin, Berlin, Germany; 2 Department of Anesthesiology and Intensive Care Medicine, Charité – Universitätsmedizin Berlin, Corporate Member of Freie Universität Berlin and Humboldt-Universität zu Berlin, Berlin, Germany

**Keywords:** Alarm management, Alarm thresholds, Intensive care unit, Artificial intelligence

## Abstract

**Background:**

High rates of clinical alarms in the intensive care unit can result in alarm fatigue among staff. Individualization of alarm thresholds is regarded as one measure to reduce non-actionable alarms. The aim of this study was to investigate staff’s perceptions of alarm threshold individualization according to patient characteristics and disease status.

**Methods:**

This is a cross-sectional survey study (February-July 2020). Intensive care nurses and physicians were sampled by convenience. Data was collected using an online questionnaire.

**Results:**

Staff view the individualization of alarm thresholds in the monitoring of vital signs as important. The extent to which alarm thresholds are adapted from the normal range varies depending on the vital sign monitored, the reason for clinical deterioration, and the professional group asked. Vital signs used for hemodynamic monitoring (heart rate and blood pressure) were most subject to alarm individualizations. Staff are ambivalent regarding the integration of novel technological features into alarm management.

**Conclusions:**

All relevant stakeholders, including clinicians, hospital management, and industry, must collaborate to establish a “standard for individualization,” moving away from ad hoc alarm management to an intelligent, data-driven alarm management. Making alarms meaningful and trustworthy again has the potential to mitigate alarm fatigue – a major cause of stress in clinical staff and considerable hazard to patient safety.

**Trial registration:**

The study was registered at ClinicalTrials.gov (NCT03514173) on 02/05/2018.

**Supplementary Information:**

The online version contains supplementary material available at 10.1186/s12913-023-09688-x.

## Background

Monitoring patients’ vital signs is part of the standard of care in the intensive care unit (ICU) [1]. Despite its proven benefits for patient safety, the incremental rise in monitoring technology over the decades has resulted in an alarm burden associated with serious risks and drawbacks for both patients and ICU staff [2–7].

Clinical alarms involve a complex interplay of social and technical factors. Highly variable and often ad hoc alarm management can result in alarms being perceived as a nuisance. Studies report up to 99% of alarms are either non-actionable or false [8]. Non-actionable alarms do not require an intervention by ICU staff. Although non-actionable alarms are valid in their technical measurement, false alarms are technically incorrect and known as artifacts. These often arise in the context of manipulation by clinical staff, including endotracheal suction, oral care, or change of a patient’s position [9]. Inappropriately set threshold limits are another leading cause for enormously high alarm rates [5]. It is common for these to remain unchanged from the manufacturer’s preset limits or for them to be adopted from the previous shift [5]. Adaptation to a patient’s current condition primarily takes place if there is a complication or a change in the severity of a patient’s condition, or upon (re)admission to the ICU after an intervention [10].

In order to maximize patient safety and reduce alarm fatigue, current recommendations recommend clinicians to individualize alarm thresholds deemed appropriate for a given patient’s state of illness and specific characteristics [5, 6, 11–13].

### Aim

The aim of this study was to investigate to what extent ICU staff’s perceptions of alarm threshold individualization vary according to patient characteristics and disease status. Furthermore, we investigated how this relates to disease severity and how medical personnel value the importance of implementing new technological features, e.g., alarm profiles and artificial intelligence (AI), in alarm management.

## Methods

### Ethics approval, consent to participate, and trial registration

Ethical approval was obtained from the Ethics Committee of the Charité – Universitätsmedizin Berlin (EA 1/031/18). Study participants were enrolled on a voluntary basis and provided written informed consent. The study was registered at ClinicalTrials.gov (NCT03514173) on 02/05/2018.

### Study setting

This study was conducted between February and July 2020 and surveyed ICU staff from two surgical ICUs of a large German academic hospital. The primary patient monitoring system used at the time of the study was the Philips IntelliVue (Koninklijke Philips NV; MX800 software version M.00.03; MMS X2 software version H.15.41-M.00.04).

### Study design

We conducted a cross-sectional survey study using an online questionnaire developed by an interdisciplinary research team, including four experienced physicians, one nurse with extensive experience in intensive care, and a computer scientist [14, 15]. Item generation was based on interdisciplinary focus group discussions, consisting of eight senior physicians and nurses with extensive ICU experience, in which topics were clustered into five sections.

The survey assessed preferences towards individualization of alarm thresholds regarding the alarm parameters heart rate (HR), systolic blood pressure (BP), peripheral oxygen saturation (SpO_2_), body temperature, and capnometry (etCO_2_). It is comprised of several sections (Table 1). Section 0 captured consent (item 1), the profession (item 2–3), and age category (item 87). In Sect. 1, participants’ views on the influence of patient characteristics — including age, pre-existing conditions, reason for admission to ICU (e.g., bleeding, sepsis), laboratory values, and medication — on setting alarm thresholds was assessed (items 4–33). Participants were asked to give one answer per patient characteristic on a predetermined, ordinal 5-point Likert scale [16]. The scale was coded as follows: “Strongly disagree” [option 1], “Disagree” [option 2], “Undecided” [option 3], “Agree” [option 4], and “Strongly agree” [option 5]. In Sect. 2, the questionnaire assessed preferred alarm thresholds, i.e., variance in values of a given alarm parameter (e.g., < 85% SpO_2_) at which ICU staff shall be immediately and urgently alarmed in the context of different diagnoses, namely chronic obstructive pulmonary disease (COPD), congestive heart failure, post generalized seizure, polytrauma, post cardiopulmonary resuscitation (CPR), and sepsis (items 34–68). This comparison of alarm thresholds between different diagnoses contained individualized 5-point levels for each alarm parameter, e.g., for HR, “<30/min” [option 1], “<35/min” [option 2], “<40/min” [option 3], “<45/min” [option 4], “<50/min” [option 5]. Other items investigated preferences towards integration of alarm profiles and individualized volume based on disease severity (Sect. 3, items 69–72), use of AI in monitoring (Sect. 4, items 73–82), and general technological affinity through the Affinity for Technology Interaction (ATI) Scale [17] (Sect. 5, items 83–86). Answers for items 69–82 could also be ranked on a Likert scale while the ATI Scale is comprised of six possible ordinal answers [17]. At the end of each item block, free-text answers were possible.

**Table 1:** Questionnaire section, item numbers, content, and example question/statement

Section	Item number	Content	Example Question/Statement
0	1–3, 87	Consent, profession, age category	*Which professional group do you belong to?*
1	4–33	Influence of patient characteristics on setting alarm thresholds	*To what extent does age play a major role in the alarm limit setting for the heart rate?*
2	34–68	Preferred alarm thresholds in acute deterioration	*At which systolic blood pressure would you like to be immediately and urgently informed for a patient with respiratory insufficiency in COPD?*
3	69–72	Alarm profiles, volume, and clinical decision support system	*Based on the patient’s disease severity, how important is it to you that alarms sound louder of softer?*
4	73–82	Artificial intelligence in setting alarm thresholds	*What advantages do you see when artificial intelligence creates patient-specific alarm profiles (e.g., for COPD) and continuously re-evaluates them?*
5	83–86	Affinity for Technology Interaction	*I like to occupy myself in greater detail with technical systems.*

Pretests among research colleagues were conducted and yielded refinement in the wording and content of questionnaire items while preserving each topic domain (Table 1). Subsequently, pilot tests among 15 members of ICU staff served to assess order, importance, and unambiguity of all survey items. Focus groups comprised of intensive care clinicians served to discuss each item’s face validity and comprehensibility as well as each target domain’s completeness. The final survey was comprised of 87 items. The full survey is available in the Additional Files.

### Data collection

A total of 253 ICU nurses and physicians working in at least one of the included ICUs was identified as potential study participants. All members of staff in the respective ICU received an invitation to complete the survey via email including detailed information about the study and a link to the online questionnaire. Over a period of 6 months, between February and July 2020, participants were able to complete the survey. At intervals of four weeks, staff received reminder emails to boost the response rate. Additionally, small flyers containing key aspects of the study, the web link, and quick response (QR) code to the online survey were distributed among ICU staff. Incentivizing study participation, a train ticket voucher worth 50€ was raffled among the participants. All study data was collected via REDCap [18, 19] and locally handled and stored on our hospital’s servers. Individuals that worked less than two days per month in the ICU were excluded from the study. The anonymized dataset is available in the Additional Files.

### Data analysis

Data analysis was conducted using Microsoft Excel [20] and R (Project for Statistical Computing) using packages including tidyverse, infer, ggplot, boot, cohen, and likert [21]. The number of free-text answers was negligible, and these were henceforth excluded in the analysis of this study.

We report the observed median for each of the 5-point survey items in Sects. 1–4 for all answers and by profession (i.e., nurse or physician). For each item in Sects. 1, 3, and 4, we also applied a bootstrap resampling procedure to quantify the sample’s variance and make more robust inferences about the population parameter [22, 23]. For each survey item, we created 15,000 bootstrap samples and calculated the respective 95% confidence intervals (CIs) from bootstrapped medians. 15,000 samples per items were deemed appropriate weighing up the robustness of the resampling approach and available computational power. Results were considered statistically significant if the median and CI excluded answer option 3 (“Undecided”). As an “Undecided” response indicates a lack of preference or directionality, if the median and CI fall entirely above or below, one can assume respondents have a significant preference [24]. A *p* value of < 0.05 was considered statistically significant.

For Sect. 2, we report the interquartile range (IQR) and investigate preferences in setting alarm thresholds for six patient scenarios with different reasons for sudden critical deterioration (Table 2). As our data is paired (each participant gives an answer for each deterioration reason) and not assumed to be normally distributed, we conducted Friedman tests to test for rank variance between patient scenarios individually for each alarm type (HR, BP, SpO_2_, body temperature, etCO_2_). Level of confidence was set at *p* < 0.05.

**Table 2:** Patient scenarios with reason for acute deterioration and respective abbreviation/acronym (Sect. 2)

Deteriorating patient scenario	Abbreviation/acronym
Patient in respiratory failure with chronic obstructive pulmonary disease	COPD
Patient with catecholamine-requiring heart failure	CHD
Patient after generalized seizure	Seizure
Patient with polytrauma	Trauma
Patient after cardiopulmonary resuscitation	Post-CPR
Septic patient	Sepsis

CHD = congestive heart disease; COPD: chronic obstructive pulmonary disease; CPR = cardiopulmonary resuscitation

In post hoc analysis, for alarm types with statistically significant results to the Friedman test, Wilcoxon signed-rank tests for matched samples were applied for each pair of the six patient scenarios. An adjusted significance level (*p* < 0.003 after Bonferroni correction where n = 15) was applied. To test for significant differences between professional groups (physicians vs. nurses) Mann–Whitney *U* tests were conducted as the two groups are independent and the data not assumed to be normally distributed. We report the *p* value, Cohen’s d, and effect magnitude as levelled by Cohen (|d|<0.2 ≙ “negligible”, |d|<0.5 ≙ “small”, |d|<0.8 ≙ “medium”, otherwise ≙ “large”) [25]. A *p* value of < 0.05 was considered statistically significant. For ATI, mean and standard deviation were calculated. Cronbach alpha was computed as reliability analysis.

## Results

Overall, 253 potential study participants were approached. Recorded answers of participants that dropped out in later stages of answering were still included in the analysis to maximize the number of responses per item. All analysis results are available in.

### Section 0: consent, age category, and profession

The response rate was 37.9% (n = 96). Due to n = 7 responses with missing consent and another n = 16 dropouts during the survey, the true response rate was 28.9% (n = 73). The studied population had a balanced representation of professional groups (n = 51 nurses, n = 35 physicians). The dominant age groups of participants were between 25 and 34 years and between 35 and 44 years, representing 38.9% (n = 28) and 40.3% (n = 29) of the studied individuals, respectively.

### Section 1: influence of patient characteristics on setting alarm thresholds

Depending on the vital sign monitored, respondents exhibited varying agreement to certain patient characteristics playing a role in adjusting the alarm thresholds (Table 3).

**Table 3:** **Perspectives on the influence of patient characteristics on alarm limits**

	Heart rate (Fig. 1)	Systolic blood pressure (Fig. 2)	SpO_2_ (Fig. 3)	Capnometry (Fig. 4)	Body temperature (Fig. 5)
Age	* 4 [ 4 ; 5 ]	* 4 [ 4 ; 5 ]	4 [ 3 ; 4 ]	3 [ 3 ; 4 ]	3 [ 3 ; 4 ]
Pre-existing conditions	* 4 [ 4 ; 4 ]	* 4 [ 4 ; 5 ]	* 5 [ 4 ; 5 ]	* 5 [ 4 ; 5 ]	3 [ 2 ; 3 ]
Reason for ICU admission	* 4 [ 4 ; 5 ]	* 5 [ 4 ; 5 ]	* 4 [ 4 ; 5 ]	* 4 [ 4 ; 5 ]	* 5 [ 4 ; 5 ]
Laboratory values	3 [ 3 ; 4 ]	3 [ 3 ; 3 ]	3 [ 3 ; 3 ]	3 [ 3 ; 4 ]	3 [ 2 ; 3 ]
Medication	* 4 [ 4 ; 5 ]	* 5 [ 4 ; 5 ]	3 [ 3 ; 4 ]	3 [ 3 ; 4 ]	3 [ 3 ; 3 ]

Observed median of 5-point Likert scale and 95% bootstrap confidence interval. Statistically significant results are marked with an asterisk (*) (*p* < 0.05). 1 = “Strongly disagree”, 2 = “Disagree”, 3 = “Undecided”, 4 = “Agree”, 5 = “Strongly agree”. ICU = intensive care unit.

**Fig. 1:**
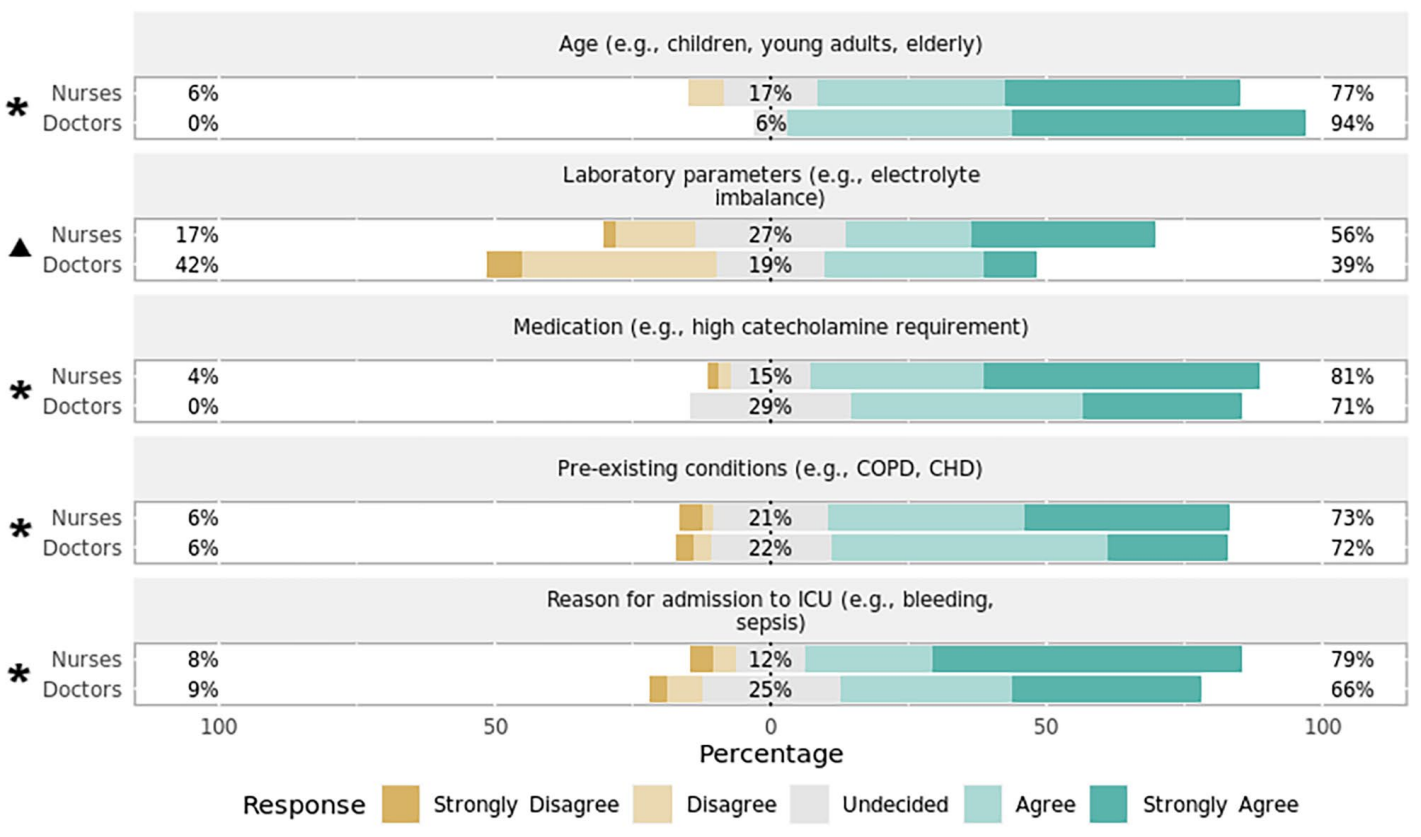
Preferences regarding setting alarm thresholds for heart rate by profession Percentages indicate the proportions of answers given per Likert item (e.g., “Agree”) by professional group. Responses marked with an asterisk (✱) are statistically significant regarding the observed median and 95% confidence interval of all responses using bootstrap resampling procedure (*p* < 0.05). Responses marked with a triangle (▲) reveal a statistically significant difference in responses stratified by profession (nurses, doctors) using Mann–Whitney *U* test (*p* < 0.05). CHD = congestive heart disease; COPD = chronic obstructive pulmonary disease; ICU = intensive care unit.

**Fig. 2:**
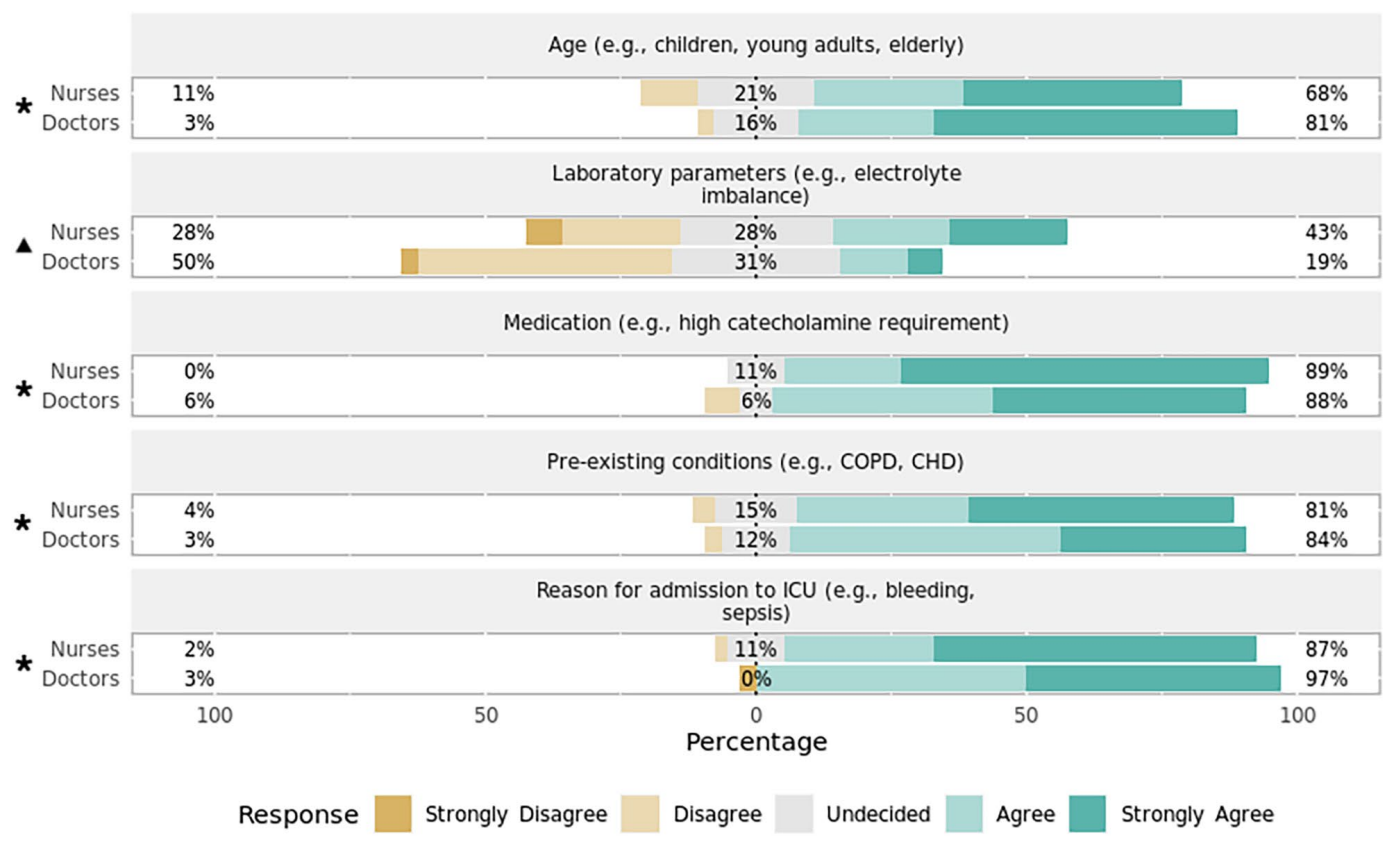
Preferences regarding setting alarm thresholds for systolic blood pressure by profession Percentages indicate the proportions of answers given per Likert item (e.g., “Agree”) by professional group. Responses marked with an asterisk (✱) are statistically significant regarding the observed median and 95% confidence interval of all responses using bootstrap resampling procedure (*p* < 0.05). Responses marked with a triangle (▲) reveal a statistically significant difference in responses stratified by profession (nurses, doctors) using Mann–Whitney *U* test (*p* < 0.05). CHD = congestive heart disease; COPD = chronic obstructive pulmonary disease; ICU = intensive care unit.

**Fig. 3:**
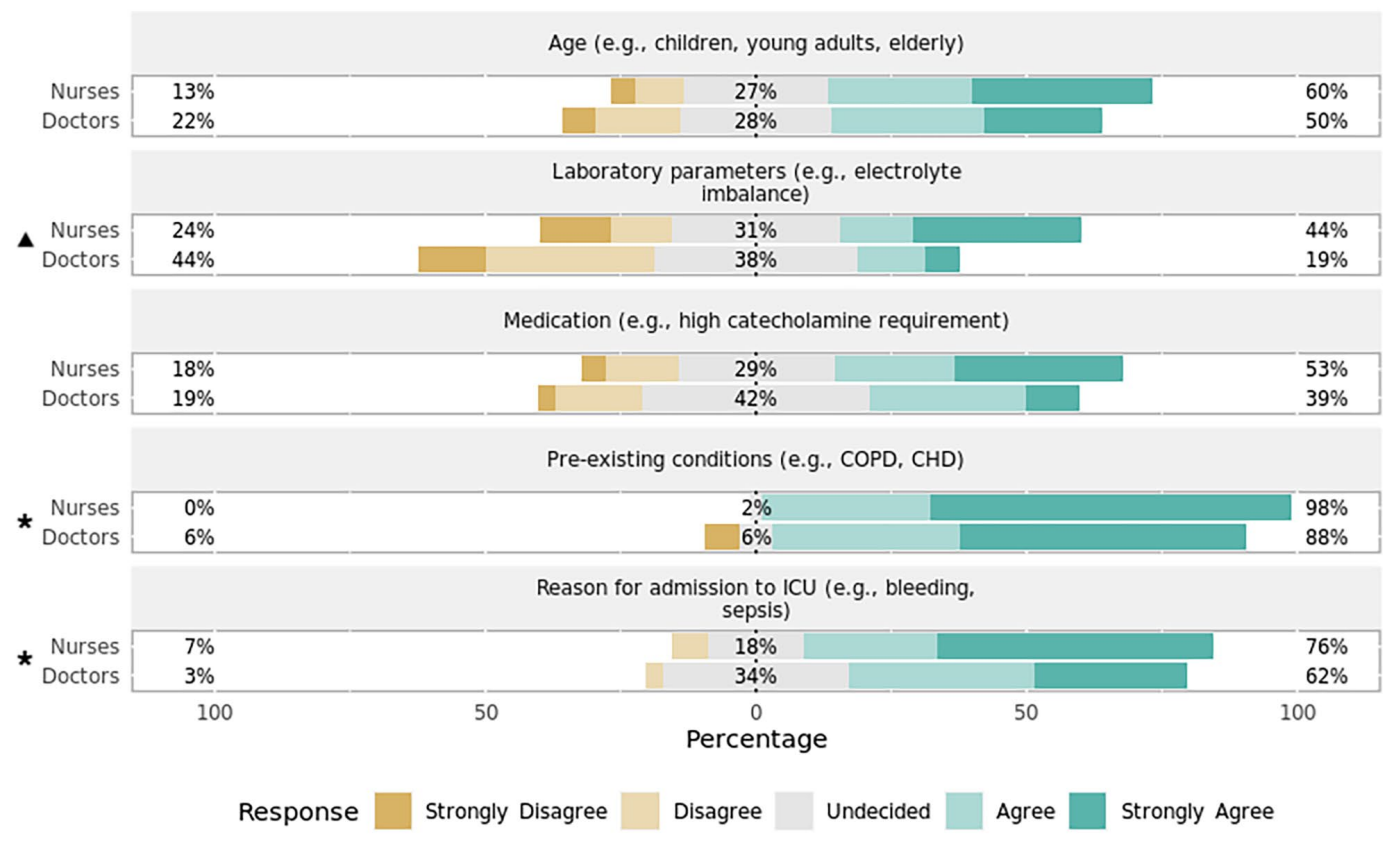
Preferences regarding setting alarm thresholds for SpO_2_ by profession Percentages indicate the proportions of answers given per Likert item (e.g., “Agree”) by professional group. Responses marked with an asterisk (✱) are statistically significant regarding the observed median and 95% confidence interval of all responses using bootstrap resampling procedure (*p* < 0.05). Responses marked with a triangle (▲) reveal a statistically significant difference in responses stratified by profession (nurses, doctors) using Mann–Whitney *U* test (*p* < 0.05). CHD = congestive heart disease; COPD = chronic obstructive pulmonary disease; ICU = intensive care unit.

**Fig. 4:**
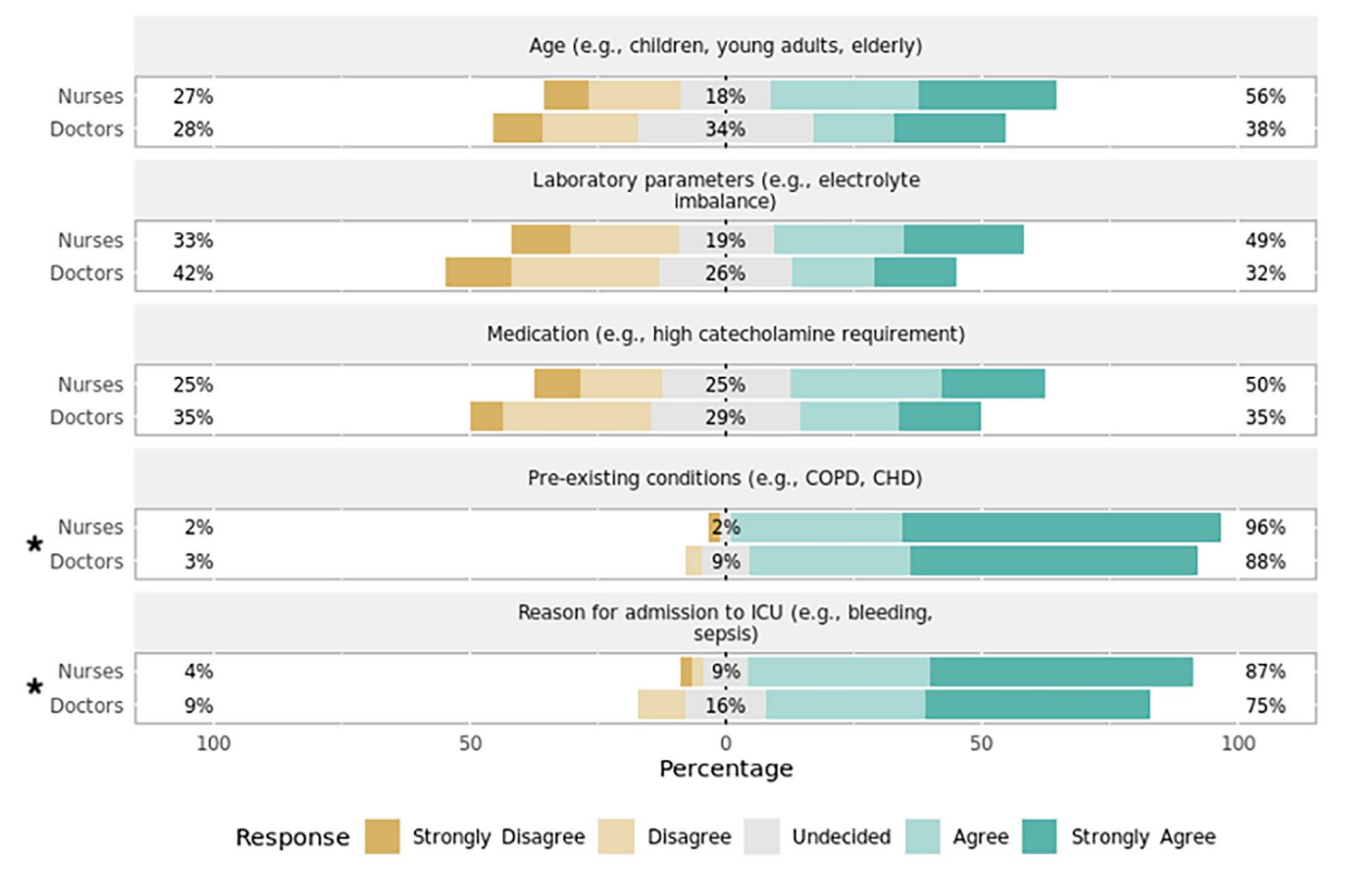
Preferences regarding setting alarm thresholds for capnometry by profession Percentages indicate the proportions of answers given per Likert item (e.g., “Agree”) by professional group. Responses marked with an asterisk (✱) are statistically significant regarding the observed median and 95% confidence interval of all responses using bootstrap resampling procedure (*p* < 0.05). Responses marked with a triangle (▲) reveal a statistically significant difference in responses stratified by profession (nurses, doctors) using Mann–Whitney *U* test (*p* < 0.05). CHD = congestive heart disease; COPD = chronic obstructive pulmonary disease; ICU = intensive care unit.

**Fig. 5:**
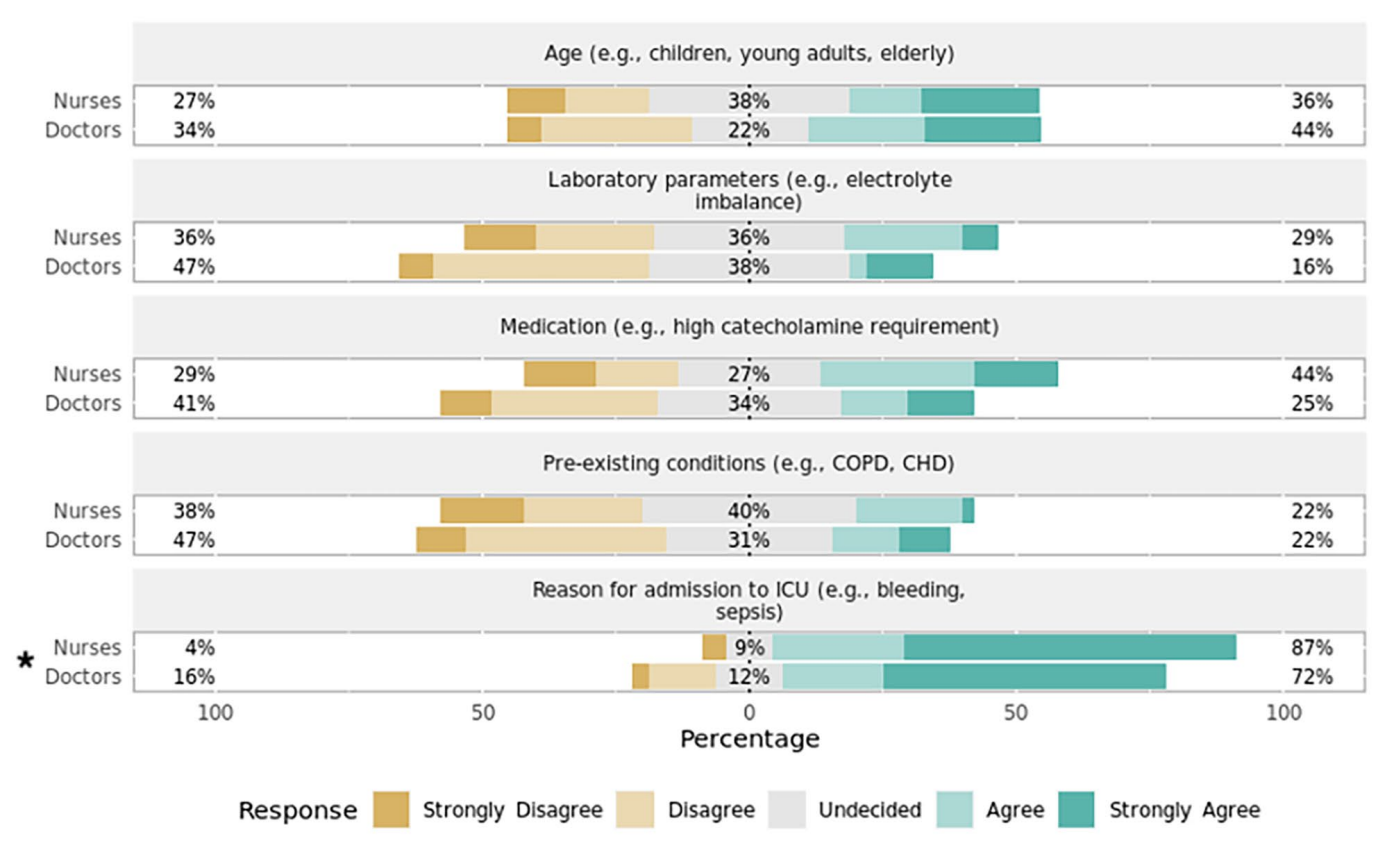
Preferences regarding setting alarm thresholds for body temperature by profession Percentages indicate the proportions of answers given per Likert item (e.g., “Agree”) by professional group. Responses marked with an asterisk (✱) are statistically significant regarding the observed median and 95% confidence interval of all responses using bootstrap resampling procedure (*p* < 0.05). Responses marked with a triangle (▲) reveal a statistically significant difference in responses stratified by profession (nurses, doctors) using Mann–Whitney *U* test (*p* < 0.05). CHD = congestive heart disease; COPD = chronic obstructive pulmonary disease; ICU = intensive care unit.

Most respondents agreed that age, pre-existing conditions, reason for ICU admission, and the administered medication play a significant role in setting the alarm limits for both HR (Fig. 1) and systolic BP (Fig. 2). When asked about threshold determination for setting alarm limits of SpO_2_ (Fig. 3) and capnometry (Fig. 4), only pre-existing conditions and the reason for ICU admission were viewed as significant characteristics. As for the monitoring of body temperature (Fig. 5), the reason for ICU admission was regarded as the single most important characteristic in determining alarm thresholds. Overall, the reason for ICU admission was the only criterion rated significant in setting alarm limits across all surveyed parameters. Conversely, ICU staff were mostly undecided regarding the importance of laboratory results for setting alarm limits.

.

#### Variation by profession

There was a statistically significant difference in how the setting of alarm thresholds is viewed by profession for HR (*p* = 0.01, effect size = 0.62, effect magnitude = 3; Fig. 1), systolic BP (*p* = 0.03, effect size = 0.52, effect magnitude = 3; Fig. 2), and SpO_2_ (*p* = 0.02, effect size = 0.55, effect magnitude = 3; Fig. 3). Nursing personnel considered laboratory values as more important across these three alarm parameters than did physicians. However, subgroup analysis did not yield a statistically significant result regarding subgroup distributions.

### Section 2: sensitivity of alarm thresholds in acute deterioration

Assuming a ICU patient scenarios (see Table 2) is acutely deteriorating, but without relevant secondary diagnoses and all other vital parameters stable, the study participants were asked at which thresholds they would like to be alarmed immediately and urgently. There was a statistically significant difference in preferences by ICU staff to be alerted for six different reasons of sudden critical deterioration across all alarm parameters assessed (HR *p* < 0.001, BP *p* < 0.001, SpO_2_*p* < 0.001, body temperature *p* < 0.001, etCO2 *p* < 0.001, Table 4).

**Table 4:** Staff preferences for alarm threshold settings of all alarm parameters assessed across six reasons for acute deterioration

	Heart rate (bpm)	Systolic blood pressure (mm Hg)	SpO_2_ (%)	Body temperature (°C)	Capnometry (mm Hg)
COPD	45 [40;50]	90 [85;90]	88 [85;90]	38.3 [38.0;38.5]	55 [50;60]
CHD	50 [45;50]	90 [85;100]	90 [89.5;92]	38.0 [38.0;38.5]	45 [45;50]
Seizure	45 [40;50]	90 [85;91.25]	90 [90;92]	38.0 [38.0;38.5]	45 [45;50]
Trauma	45 [40;50]	90 [85;95]	91.5 [90;92]	38.0 [38.0;38.5]	45 [45;50]
Post-CPR	50 [45;50]	90 [88.75;100]	92 [90;92]	38.0 [37.8;38.0]	45 [45;50]
Sepsis	50 [45;50]	90 [88.75;100]	90 [90;92]	38.0 [38.0;38.5]	45 [45;50]
*P* value	< 0.001	< 0.001	< 0.001	< 0.001	< 0.001

Median [IQR] of each alarm parameter threshold rated by reason for critical deterioration. *P* values of the Friedman tests comparing preferences to be alerted for six different reasons of sudden critical deterioration across all alarm parameters assessed. Level of significance was at *p* < 0.05. CHD = congestive heart disease; COPD = chronic obstructive pulmonary disease; CPR = cardiopulmonary resuscitation.

Table 5 shows the reciprocal comparison of the six patient scenarios listed in Table 2. There were statistically significant differences with large effect magnitudes at which thresholds ICU staff wanted to be alerted for SpO_2_ and capnometry regarding patients with COPD in acute respiratory failure as compared to all other deterioration reasons. Median SpO_2_ was 88% [85;90] and median etCO_2_ was 55mmHg [50;60]. There was a similar pattern with small to medium effect sizes in the alarm limits of HR (45 bpm [40;50]) and systolic BP (90mmHg [85;90]) for the patient scenario COPD. Preferences in setting alarm limits for body temperature in the patient scenario post-CPR were also significantly different from those for all other reasons for deterioration with medium to large effect magnitudes. Overall, the hemodynamically relevant alarm parameters (HR and BP) had the highest number of significant individual adjustments across different patient scenarios.

**Table 5:** Comparison of different deterioration reasons on perspectives for setting alarm thresholds

		COPD	CHD	Seizure	Trauma	Post-CPR
Heart rate	CHD	< 0.001 ^b^	*N/A*	< 0.001 ^a^	0.034	0.020
	Seizure	0.193	< 0.001 ^a^	*N/A*	0.071	< 0.001 ^b^
	Trauma	0.004	0.034	0.071	* N/A*	< 0.001 ^a^
	Post-CPR	< 0.001 ^b^	0.020	< 0.001 ^b^	< 0.001 ^a^	*N/A*
	Sepsis	< 0.001 ^b^	0.119	< 0.001 ^b^	0.001 ^a^	0.599
Systolic blood pressure	CHD	< 0.001 ^a^	*N/A*	0.003 ^a^	0.552	0.163
	Seizure	0.124	0.003 ^a^	*N/A*	0.020	< 0.001 ^a^
	Trauma	< 0.001 ^a^	0.552	0.020	* N/A*	0.026
	Post-CPR	< 0.001 ^b^	0.163	< 0.001 ^a^	0.026	* N/A*
	Sepsis	< 0.001 ^a^	0.676	0.005	0.329	0.205
SpO_2_	CHD	< 0.001 ^c^	*N/A*	0.170	0.004	0.001 ^b^
	Seizure	< 0.001 ^c^	0.170	* N/A*	0.063	0.093
	Trauma	< 0.001 ^c^	0.004	0.063	* N/A*	0.744
	Post-CPR	< 0.001 ^c^	0.001 ^b^	0.093	0.744	* N/A*
	Sepsis	< 0.001 ^c^	0.009	0.460	0.447	0.144
Body temperature	CHD	0.019	* N/A*	0.531	0.213	< 0.001 ^b^
	Seizure	0.330	0.531	* N/A*	0.016	< 0.001 ^b^
	Trauma	0.014	0.213	0.016	* N/A*	< 0.001 ^b^
	Post-CPR	< 0.001 ^c^	< 0.001 ^b^	< 0.001 ^b^	< 0.001 ^b^	*N/A*
	Sepsis	0.029	0.477	0.263	0.426	< 0.001 ^b^
Capnometry	CHD	< 0.001 ^c^	*N/A*	0.864	0.073	0.093
	Seizure	< 0.001 ^c^	0.864	* N/A*	0.028	0.164
	Trauma	< 0.001 ^c^	0.073	0.028	* N/A*	0.688
	Post-CPR	< 0.001 ^c^	0.093	0.164	0.688	* N/A*
	Sepsis	< 0.001 ^c^	0.279	0.383	0.395	0.507

*P* values of Wilcoxon signed-rank test with *p* < 0.003 (Bonferroni correction with n = 15) for the comparison of different deterioration reasons for alarm thresholds of SpO_2_, capnometry, heart rate, systolic blood pressure, and body temperature. ^a^ corresponds to small effect magnitude (|d|<0.5 ≙ “small”), ^b^ to medium effect magnitude (|d|<0.8 ≙ “medium”), and ^c^ to large effect magnitude (otherwise ≙ “large”). CHD = congestive heart disease; COPD = chronic obstructive pulmonary disease; CPR = cardiopulmonary resuscitation

#### Variation by profession

**Fig. 6:**
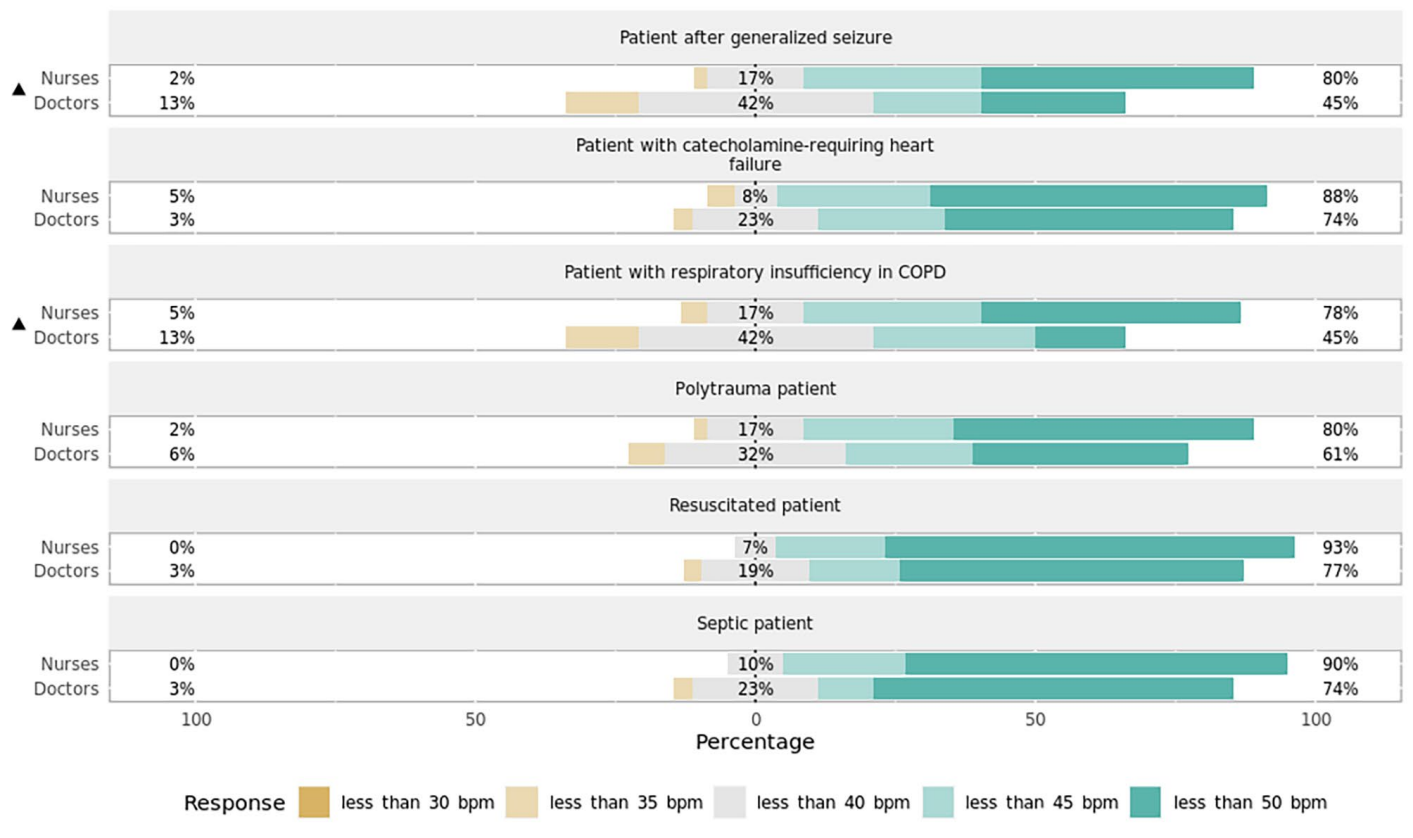
Preferences in setting heart rate alarm thresholds depending on reason for deterioration by profession Percentages indicate the proportions of answers given per Likert item (e.g., “Agree”) by professional group. Responses marked with an asterisk (✱) are statistically significant regarding the observed median and 95% confidence interval of all responses using bootstrap resampling procedure. Responses marked with a triangle (▲) reveal a statistically significant difference in responses stratified by profession (nurses, doctors) using Mann–Whitney *U* test (*p* < 0.05). COPD = chronic obstructive pulmonary disease.

There was a statistically significant difference in how nursing personnel preferred to set the sensitivity of the HR lower alarm threshold for the patient scenarios COPD (*p* = 0.002, effect size = 0.78, effect magnitude = 3; Fig. 6) and seizure (*p* = 0.004, effect size = 0.75, effect magnitude = 3; Fig. 6), as opposed to all other reasons of deterioration. All other alarm parameters did not vary significantly between nurses and physicians.

### Section 3: alarm profiles, volume, and clinical decision support system

**Fig. 7:**
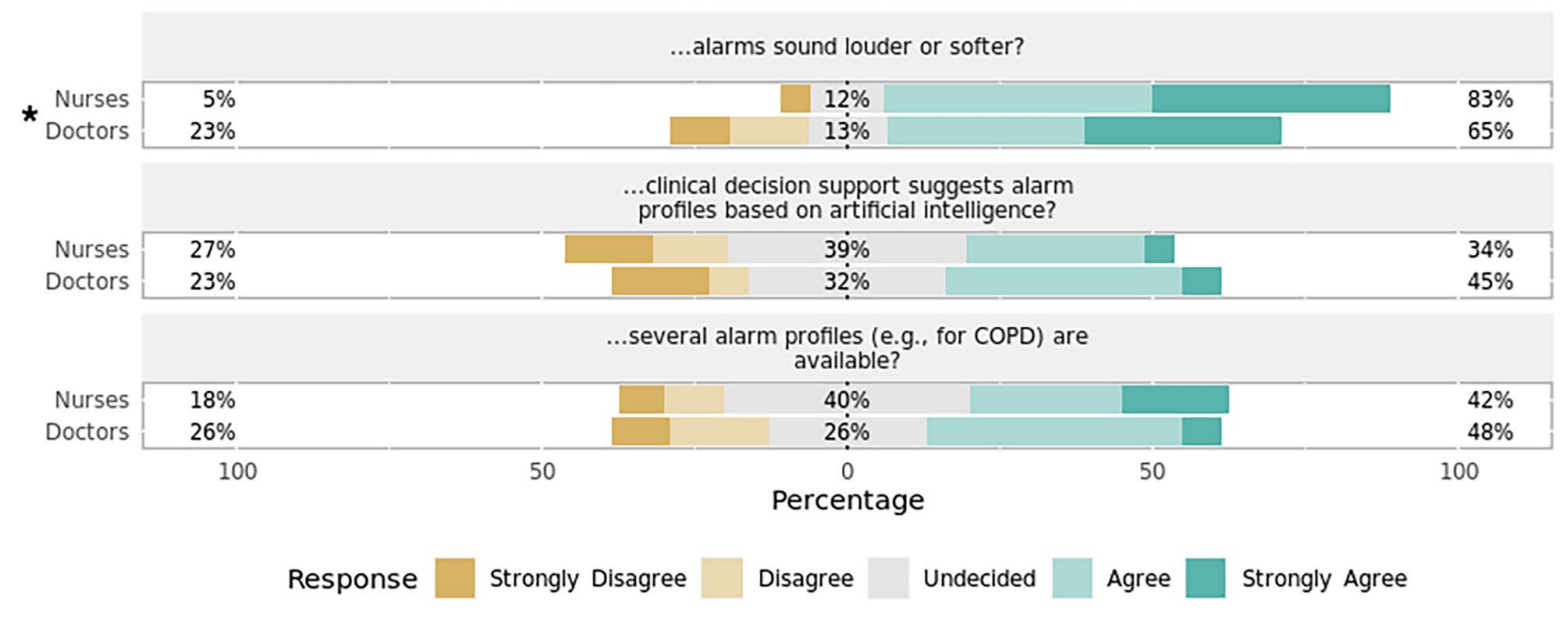
Importance of alarm profiles, volume, and clinical decision support system in setting alarms by profession Respondents’ answers to the question, “Based on the patient’s disease severity, how important is it to you that…”. Percentages indicate the proportions of answers given per Likert item (e.g., “Agree”) by professional group. Responses marked with an asterisk (✱) are statistically significant regarding the observed median and 95% confidence interval of all responses using bootstrap resampling procedure. Responses marked with a triangle (▲) reveal a statistically significant difference in responses stratified by profession (nurses, doctors) using Mann–Whitney *U* test (*p* < 0.05). COPD = chronic obstructive pulmonary disease.

Staff considered it essential that the alarm volume should be adapted to the criticality of the patient’s condition (Fig. 7). Opinions on clinical decision support systems (CDSS) suggesting alarm profiles based on AI or the availability of alarm profiles for certain conditions (e.g., COPD) were mostly split (Fig. 7). There was no statistically significant difference in answers between nurses and physicians.

### Section 4: perceived AI in setting alarm thresholds

**Fig. 8:**
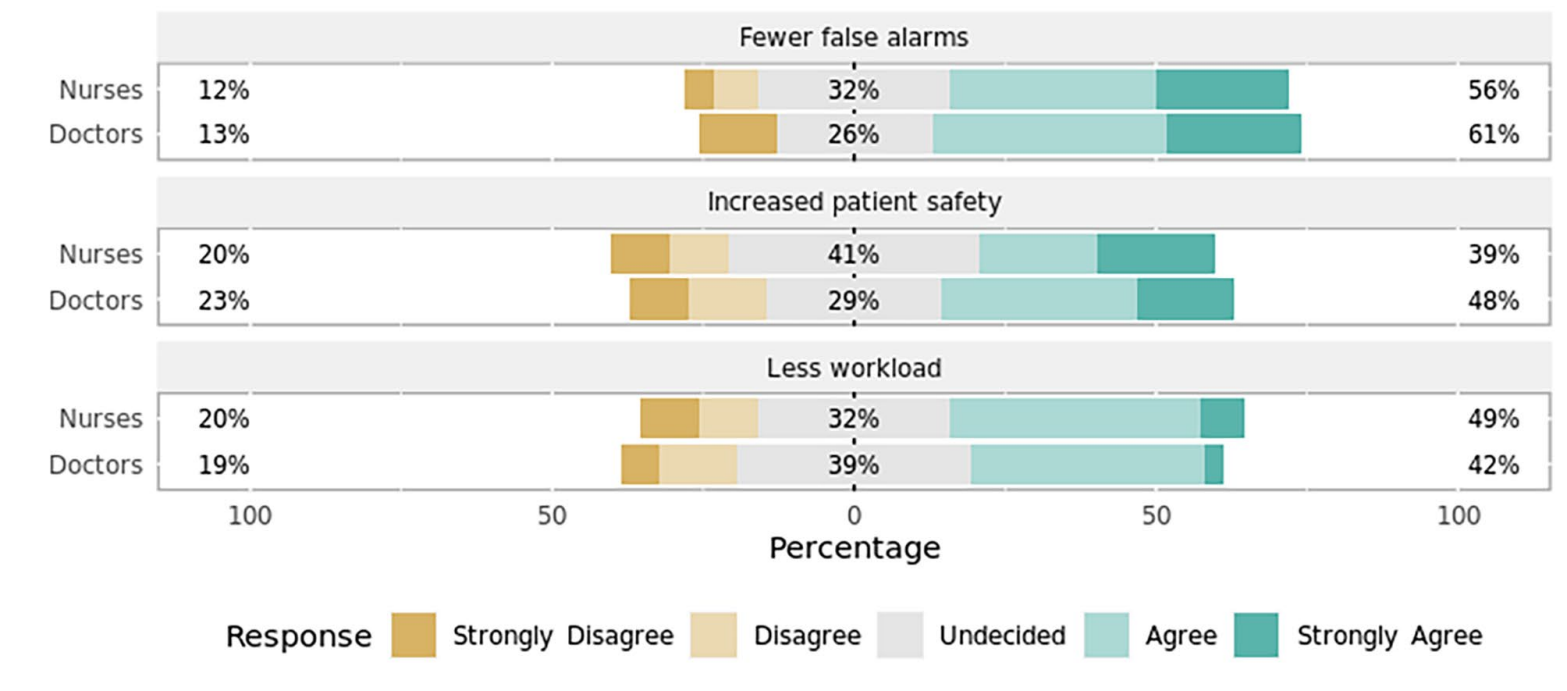
Advantages of artificial intelligence in setting alarm thresholds by profession Respondents’ answers to the question, “What advantages do you see when artificial intelligence suggests alarm profiles (e.g., for COPD) on a patient-specific basis and continuously reevaluates them?” Percentages indicate the proportions of answers given per Likert item (e.g., “Agree”) by professional group. Responses marked with an asterisk (✱) are statistically significant regarding the observed median and 95% confidence interval of all responses using bootstrap resampling procedure. Responses marked with a triangle (▲) reveal a statistically significant difference in responses stratified by profession (nurses, doctors) using Mann–Whitney *U* test (*p* < 0.05).

**Fig. 9:**
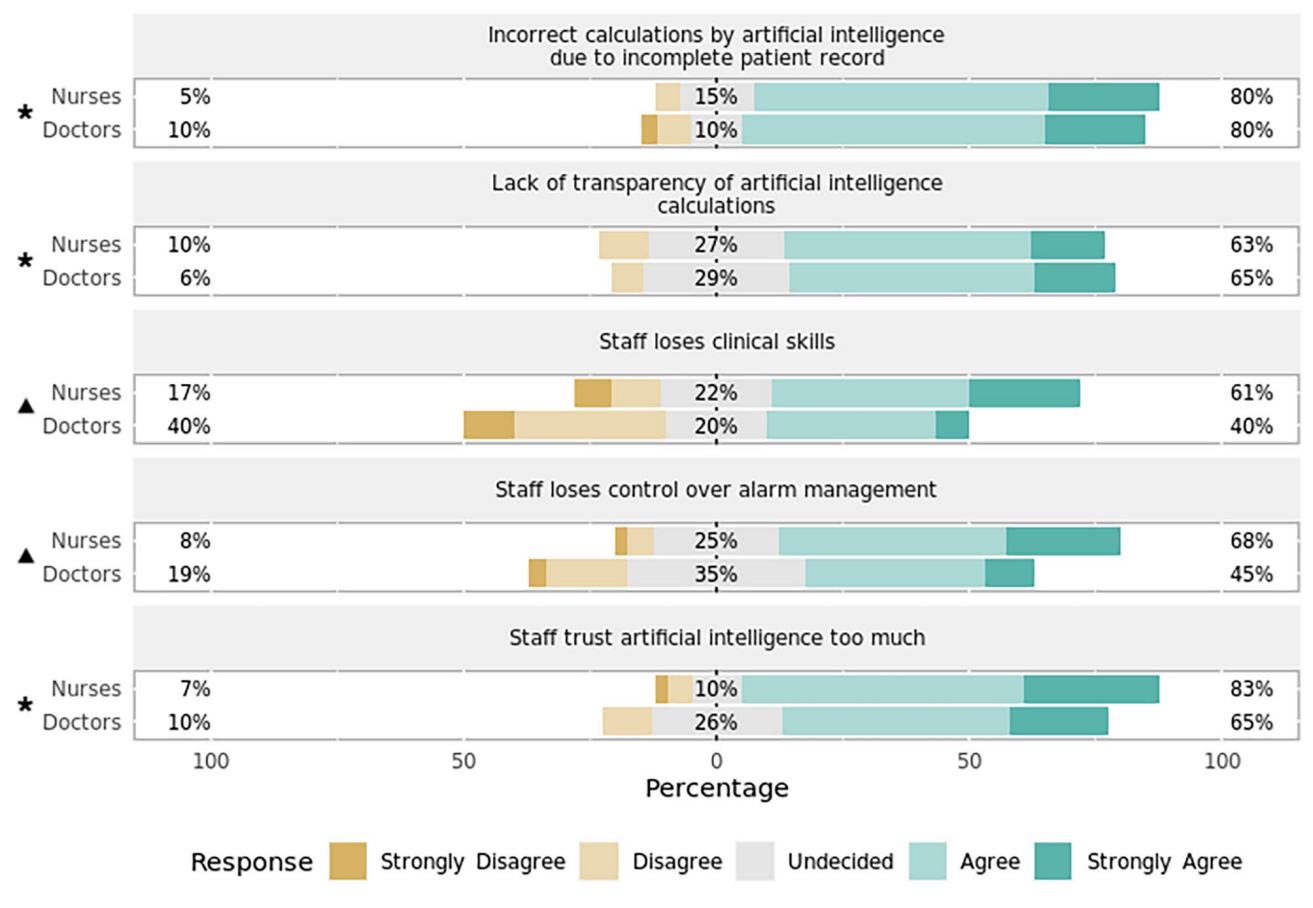
Perceived dangers of artificial intelligence in setting alarm thresholds by profession Respondents’ answers to the question, “What dangers do you see when artificial intelligence suggests alarm profiles (e.g., for COPD) on a patient-specific basis and continuously reevaluates them?” Percentages indicate the proportions of answers given per Likert item (e.g., “Agree”) by professional group. Responses marked with an asterisk (✱) are statistically significant regarding the observed median and 95% confidence interval of all responses using bootstrap resampling procedure. Responses marked with a triangle (▲) reveal a statistically significant difference in responses stratified by profession (nurses, doctors) using Mann–Whitney *U* test (*p* < 0.05).

Given an AI would suggest and continuously reevaluate alarm profiles on a patient-specific basis, most ICU staff regarded this to be advantageous in reducing the number of false alarms (Fig. 8). Views were split regarding the potential of AI-suggested alarm limits leading to an increase in patient safety or a decrease in workload for staff (Fig. 8). Concerns included flawed algorithms due to incomplete patient records, insufficient transparency of calculations, and an unwarranted trust in AI (Fig. 9). Nursing staff were also significantly more concerned about the loss of clinical skills (*p* = 0.03, effect size = 0.53, effect magnitude = 3) and a loss of control over alarm management (*p* = 0.04, effect size = 0.50, effect magnitude = 2) following the introduction of intelligent alarm profiles (Fig. 9). For the latter item, subgroup analysis yielded a statistically significant result among the surveyed ICU nurses in favor of the concern.

### Section 5: ATI scale

Participants’ self-reported technological affinity was on average 4.2 (SD 1.1), the maximum of 6.0 representing extraordinarily strong technological affinity. Cronbach alpha was on average 0.85 (95% CI [0.8–0.9]), representing a good internal consistency reliability [26].

## Discussion

### Principal findings

This study generated five principal findings by surveying intensive care unit staff:

1. Staff considered the reason for ICU admission as greatest contributor in setting and adjusting appropriate alarm thresholds of all commonly measured vital signs for each patient individually.
2. Alarm limit adjustment of heart rate and systolic blood pressure were most dependent on patient characteristics (age, medication, pre-existing conditions, and reason for intensive care unit admission).
3. Threshold limits of SpO_2_ and capnometry in patients with pre-existing chronic pulmonary obstructive disease were significantly different from other patient groups.
4. Nurses valued laboratory values significantly more than physicians in determining threshold limits for systolic blood pressure, heart rate, and SpO_2_.
5. Nurses viewed the integration of intelligent alarm profiles more critically than doctors.

### Patient-specific individualization of vital sign alarm thresholds

This questionnaire’s findings confirmed our hypothesis that ICU staff regard alarm threshold individualization according to patient characteristics and criticality as a necessity. However, the degree to which patient characteristics influence the adjustment of alarm limits varied significantly among the monitored vital signs assessed. Respondents judged the adjustment of the hemodynamic parameters (HR, BP) to be subject to nearly all patient characteristics assessed. This may be ascribed to several reasons. First, medication used in critical care often directly or adversely impacts HR and BP, hence requiring custom monitoring. Second, age is an important determinant in the ability of the cardiovascular system to restore sufficient cardiac output while being much more trivial for sufficient blood gas and temperature homeostasis. Third, the reason for ICU admission and pre-existing conditions may have been important to respondents as a plethora of critically ill patients will be hemodynamically unstable upon admission due to a destabilizing current condition, a deterioration of an existing illness, or an interplay thereof. These two attributes’ alarm thresholds were significantly influenced not just by the hemodynamic parameters, but all vital signs assessed (except for pre-existing conditions in body temperature). Since it is at the core of intensive care medicine to enable and stabilize homeostasis in per se unstable patients, one may argue these two aspects are generally considered important when adjusting alarm thresholds.

The perceived importance of the admission diagnosis for alarm management also supports the observation that ICU admission is often the only constant in time focusing on the determination of alarm limits, often disregarded at later stages of an ICU stay. Previous studies suggest alarm thresholds often remain unchanged for prolonged periods of times or at factory default settings [5]. In particular, BP and HR, being highly dependent on often variable catecholamine administration, should warrant regular review of alarm thresholds. Regarding the adjustment of body temperature alarm limits, this can be considered routine in the context of pre-existing or therapeutically induced hypothermia following successful CPR [27–29]. However, despite being customary practice, a recent systematic review and meta-analysis revealed no benefit for survival or functional outcome with forms of hypothermia as compared to normothermia [30].

On a more general note, although 38 °C was widely chosen as the upper bound alarm threshold for body temperature, current evidence does not indicate fever management to have a significant effect on patient survival [31]. In light of this, clinicians may consider adjusting alarm limits so as to only result in actionable alarms, where actions ideally encompass evidence-based clinical decisions. In contrast, laboratory values were not judged as significant for the setting of alarm threshold limits. This may indicate a perceived subordinate role of laboratory abnormalities, e.g., electrolyte imbalances, for the generation of false or non-actionable alarms. Although participants were rather undecided about the significance of laboratory values for alarm thresholds, nurses judged them to be more important than doctors.

### Alarm limits in states of clinical deterioration

Current evidence suggests clinicians mostly do not adjust alarm limits before the occurrence of a complication, a change in severity of a patient’s condition, or upon (re)admission to the ICU [10]. When asked about a certain reason for deterioration, respondents chose different alarm threshold sensitivities for each vital sign monitored. In the case of COPD, indicated alarm limits were significantly lower for SpO_2_ and higher for capnometry in comparison to all other deterioration reasons assessed. This, however, can be considered standard practice as the lower end threshold of SpO_2_ of 88% corresponds to the current threshold for therapeutic consequences in pre-existing COPD [32]. It may suggest that an alarm profile for COPD patients could be beneficial as an “individualized standard,” potentially helping avoid a plethora of non-actionable SpO_2_ alarms.

### Individualization and standardization

At our institution, alarm adjustment is currently done in a non-standardized, ad hoc manner. There is a clear mismatch between individualizations already routinely done and the lack of a standard operating procedure or guideline, desired by ICU staff [33]. As much as alarm limits are individualized according to patients’ characteristics, there should be standardization in the way alarm limits are established and reviewed. Individualization may not only involve the threshold sensitivity but also other parameters. Depending on criticality, respondents indicated alarm volume should be adjusted accordingly. Previous studies also argue that alarm delays [34] or changing the alarm tones [35, 36] may also be suitable measures to establish alarm hygiene.

### Alarm profiles and the integration of AI

There are mixed views on the integration of alarm profiles based on AI, with concerns outweighing benefits according to the surveyed population. Reasons ranged from possible inaccuracies underlying the dependence of human input for accurate calculations to concerns about losing clinical skills and control. In one of our previous studies, staff gave similar arguments against AI [33]. Here, physicians and nurses deemed it essential to rely on learned competence and patient observation rather than trusting technology. In this study, nurses were significantly more concerned about the integration of AI-powered alarm profiles than doctors. Overall, this stands in contrast with previously observed positive effects on staff by an “alarm advisor.” In this study, clinicians felt relieved, perceived subjectively fewer false alarms, and readily learned to use the technology [13].

This observed skepticism towards the integration of AI-powered alarm profiles warrants not only the consideration of technical accuracy and feasibility but also staff involvement and engagement once such endeavors are planned. Before implementing innovative technology, it is imperative to address and mitigate all skepticism by staff [37].

### Limitations

Sampling of participants took place at a single site, and the response rate was moderately low. The latter might have been negatively impacted as data collection took place during the COVID-19 pandemic, which resulted in a substantial increase in workload for ICU staff. As the number of personnel asked to complete the survey was large, the total count of responses was given precedence over the response rate until considered satisfactory. Further, expert opinion does not necessarily represent what is considered best practice according to latest scientific evidence and might also not reflect real-word practice. However, considering that adherence to internationally accepted guidelines for alarm management is mostly unknown and varies by hospital setting, local practice patterns, and between countries, surveying individual opinions seemed appropriate to estimate current practice. We did not include all characteristics that could have an influence on alarm settings, and other factors are most likely involved, for example staffing. Due to exhaustiveness of the survey, we worked with examples and abstained from including structural factors. Lastly, the nature of an online survey may lead to an inclusion bias, making it more likely for tech-savvy individuals to take part.

## Conclusions

Despite wide agreement among ICU staff that alarm thresholds of standardly monitored vital signs should be individualized according to patient-specific and situational factors, current alarm routine does not address this in a standardized manner. All relevant stakeholders, including hospital management and industry, should collaborate on assessing current alarm management processes and policies. The goal is to develop a local “standard for individualization,” resulting in alarm management that is regular and structured, yet tailored to individual patient groups. Ideally, this shall rely on both subjective and objective parameters (i.e., routine clinical data) for clinical decision-making, enabling more data-driven and intelligent alarm management. Successful transformation also necessitates inclusion of differing views and requirements by professional groups as well as varying levels of technological affinity and digital literacy. Under this premise, future-proof alarm management promises to not only improve patient outcomes but also minimize the alarm burden for ICU staff.

## Electronic supplementary material

Below is the link to the electronic supplementary material.

Supplementary Material 1

Supplementary Material 2

Supplementary Material 3

Supplementary Material 4

## Data Availability

All data generated or analyzed during this study are included in this published article and its supplementary information files.

## List of Abbreviations

AI: Artificial intelligence
ATI: Affinity for Technology Interaction
BP: Blood pressure
CDSS: Clinical decision support system
CHD: Congestive heart disease
CI: Confidence interval
COPD: Chronic obstructive pulmonary disease
CPR: Cardiopulmonary resuscitation
etCO_2_: Capnometry
HR: Heart rate
ICU: Intensive care unit
IQR: Interquartile range
SpO_2_: Peripheral oxygen saturation
