# Supplementary material for: Staff perspectives on the influence of patient characteristics on alarm management in the intensive care unit: a cross-sectional survey study
Source: BMC Health Serv Res. 2023 Jul 5;23:729. doi: 10.1186/s12913-023-09688-x (PMC10324165; doi:10.1186/s12913-023-09688-x)
Supplement: Supplementary file 1 — Supplementary Material 1 [file 12913_2023_9688_MOESM1_ESM.docx]

**Additional file 1, Document (.docx): Study survey.**

**Influence of Patient Characteristics on Alarm Management in the Intensive Care Unit**

This sub-study is part of the SARPI study "Staff Acceptance of Remote Patient Monitoring on ICU." Participation is anonymous and voluntary. No consequences under employment law can be derived from the survey and you may drop out or skip questions at any time. There will be no performance or behavioral assessment. All data required for the evaluation, including personal data (age, occupation, etc.), will be stored electronically on the clinic server and destroyed after 10 years.

The Ethics Committee (EA1_031_18) and the Staff Council have approved the survey. If you have any questions, feel free to contact Prof. Dr. Dr. Felix Balzer (study director) or Dr. Akira-Sebastian Poncette (study coordinator). You can reach them via phone or email.

1. I agree that personal data may be stored, evaluated, and published anonymously within the research project mentioned above.

- Agree

1. Do you currently work regularly in intensive care (more than 2 days per month)?

- Yes
- No

1. Which professional group do you belong to?

- Physician
- Nurse

You will now be asked about several vital signs and to what extent patient characteristics (e.g., age, diagnosis) play a role in the respective alarm limit setting on the patient monitor.

**To what extent does the following patient characteristic play a major role in the alarm limit setting for the heart rate?**

1. Age (e.g., children, young adults, elderly)

- Strongly disagree
- Disagree
- Undecided
- Agree
- Strongly agree

1. Pre-existing conditions (e.g., COPD, CHD)

- Strongly disagree
- Disagree
- Undecided
- Agree
- Strongly agree

1. Reason for admission to ICU (e.g., bleeding, sepsis)

- Strongly disagree
- Disagree
- Undecided
- Agree
- Strongly agree

1. Laboratory parameters (e.g., electrolyte imbalance)

- Strongly disagree
- Disagree
- Undecided
- Agree
- Strongly agree

1. Medication (e.g., high catecholamine requirement)

- Strongly disagree
- Disagree
- Undecided
- Agree
- Strongly agree

1. Other: ______________

**The following patient characteristics play a major role in the alarm limit setting for the blood pressure.**

1. Age (e.g., children, young adults, elderly)

- Strongly disagree
- Disagree
- Undecided
- Agree
- Strongly agree

1. Pre-existing conditions (e.g., COPD, CHD)

- Strongly disagree
- Disagree
- Undecided
- Agree
- Strongly agree

1. Reason for admission to ICU (e.g., bleeding, sepsis)

- Strongly disagree
- Disagree
- Undecided
- Agree
- Strongly agree

1. Laboratory parameters (e.g., electrolyte imbalance)

- Strongly disagree
- Disagree
- Undecided
- Agree
- Strongly agree

1. Medication (e.g., high catecholamine requirement)

- Strongly disagree
- Disagree
- Undecided
- Agree
- Strongly agree

1. Other: ______________

**The following patient characteristics play a major role in the alarm limit setting for the oxygen saturation.**

1. Age (e.g., children, young adults, elderly)

- Strongly disagree
- Disagree
- Undecided
- Agree
- Strongly agree

1. Pre-existing conditions (e.g., COPD, CHD)

- Strongly disagree
- Disagree
- Undecided
- Agree
- Strongly agree

1. Reason for admission to ICU (e.g., bleeding, sepsis)

- Strongly disagree
- Disagree
- Undecided
- Agree
- Strongly agree

1. Laboratory parameters (e.g., electrolyte imbalance)

- Strongly disagree
- Disagree
- Undecided
- Agree
- Strongly agree

1. Medication (e.g., high catecholamine requirement)

- Strongly disagree
- Disagree
- Undecided
- Agree
- Strongly agree

1. Other: ______________

**The following patient characteristics play a major role in the alarm limit setting for the body temperature.**

1. Age (e.g., children, young adults, elderly)

- Strongly disagree
- Disagree
- Undecided
- Agree
- Strongly agree

1. Pre-existing conditions (e.g., COPD, CHD)

- Strongly disagree
- Disagree
- Undecided
- Agree
- Strongly agree

1. Reason for admission to ICU (e.g., bleeding, sepsis)

- Strongly disagree
- Disagree
- Undecided
- Agree
- Strongly agree

1. Laboratory parameters (e.g., electrolyte imbalance)

- Strongly disagree
- Disagree
- Undecided
- Agree
- Strongly agree

1. Medication (e.g., high catecholamine requirement)

- Strongly disagree
- Disagree
- Undecided
- Agree
- Strongly agree

1. Other: ______________

**The following patient characteristics play a major role in the alarm limit setting for the capnometry.**

1. Age (e.g., children, young adults, elderly)

- Strongly disagree
- Disagree
- Undecided
- Agree
- Strongly agree

1. Pre-existing conditions (e.g., COPD, CHD)

- Strongly disagree
- Disagree
- Undecided
- Agree
- Strongly agree

1. Reason for admission to ICU (e.g., bleeding, sepsis)

- Strongly disagree
- Disagree
- Undecided
- Agree
- Strongly agree

1. Laboratory parameters (e.g., electrolyte imbalance)

- Strongly disagree
- Disagree
- Undecided
- Agree
- Strongly agree

1. Medication (e.g., high catecholamine requirement)

- Strongly disagree
- Disagree
- Undecided
- Agree
- Strongly agree

1. Other: ______________

You are working at maximum capacity in the ICU. Below you will find questions about various vital sign values for which you would like to be immediately alarmed. The listed patients have no relevant secondary diagnoses, and all other vital signs are stable.

**At which systolic blood pressure would you like to be immediately and urgently informed for the following adult patients?**

1. Patient with respiratory insufficiency in COPD

- < 80 mmHg
- < 85 mmHg
- < 90 mmHg
- < 95 mmHg
- < 100 mmHg

1. Patient with catecholamine-requiring heart failure

- < 80 mmHg
- < 85 mmHg
- < 90 mmHg
- < 95 mmHg
- < 100 mmHg

1. Patient after generalized seizure

- < 80 mmHg
- < 85 mmHg
- < 90 mmHg
- < 95 mmHg
- < 100 mmHg

1. Polytrauma patient

- < 80 mmHg
- < 85 mmHg
- < 90 mmHg
- < 95 mmHg
- < 100 mmHg

1. Resuscitated patient

- < 80 mmHg
- < 85 mmHg
- < 90 mmHg
- < 95 mmHg
- < 100 mmHg

1. Septic patient

- < 80 mmHg
- < 85 mmHg
- < 90 mmHg
- < 95 mmHg
- < 100 mmHg

1. Other: ___________

**At which heart rate would you like to be immediately and urgently informed for the following adult patients?**

1. Patient with respiratory insufficiency in COPD

- < 30 bpm
- < 35 bpm
- < 40 bpm
- < 45 bpm
- < 50 bpm

1. Patient with catecholamine-requiring heart failure

- < 30 bpm
- < 35 bpm
- < 40 bpm
- < 45 bpm
- < 50 bpm

1. Patient after generalized seizure

- < 30 bpm
- < 35 bpm
- < 40 bpm
- < 45 bpm
- < 50 bpm

1. Polytrauma patient

- < 30 bpm
- < 35 bpm
- < 40 bpm
- < 45 bpm
- < 50 bpm

1. Resuscitated patient

- < 30 bpm
- < 35 bpm
- < 40 bpm
- < 45 bpm
- < 50 bpm

1. Septic patient

- < 30 bpm
- < 35 bpm
- < 40 bpm
- < 45 bpm
- < 50 bpm

1. Other: ___________

**At which oxygen saturation would you like to be immediately and urgently informed for the following adult patients?**

1. Patient with respiratory insufficiency in COPD

- < 83 %
- < 85 %
- < 88 %
- < 90 %
- < 92 %

1. Patient with catecholamine-requiring heart failure

- < 83 %
- < 85 %
- < 88 %
- < 90 %
- < 92 %

1. Patient after generalized seizure

- < 83 %
- < 85 %
- < 88 %
- < 90 %
- < 92 %

1. Polytrauma patient

- < 83 %
- < 85 %
- < 88 %
- < 90 %
- < 92 %

1. Resuscitated patient

- < 83 %
- < 85 %
- < 88 %
- < 90 %
- < 92 %

1. Septic patient

- < 83 %
- < 85 %
- < 88 %
- < 90 %
- < 92 %

1. Other: ___________

**At which body temperature would you like to be immediately and urgently informed for the following adult patients?**

1. Patient with respiratory insufficiency in COPD

- > 37,8°C
- > 38,0°C
- > 38,3°C
- > 38,5°C
- > 38,8°C

1. Patient with catecholamine-requiring heart failure

- > 37,8°C
- > 38,0°C
- > 38,3°C
- > 38,5°C
- > 38,8°C

1. Patient after generalized seizure

- > 37,8°C
- > 38,0°C
- > 38,3°C
- > 38,5°C
- > 38,8°C

1. Polytrauma patient

- > 37,8°C
- > 38,0°C
- > 38,3°C
- > 38,5°C
- > 38,8°C

1. Resuscitated patient

- > 37,8°C
- > 38,0°C
- > 38,3°C
- > 38,5°C
- > 38,8°C

1. Septic patient

- > 37,8°C
- > 38,0°C
- > 38,3°C
- > 38,5°C
- > 38,8°C

1. Other: ___________

**At which end-tidal CO2 would you like to be immediately and urgently informed for the following adult patients?**

1. Patient with respiratory insufficiency in COPD

- > 40 mmHg
- > 45 mmHg
- > 50 mmHg
- > 55 mmHg
- > 60 mmHg

1. Patient with catecholamine-requiring heart failure

- > 40 mmHg
- > 45 mmHg
- > 50 mmHg
- > 55 mmHg
- > 60 mmHg

1. Patient after generalized seizure

- > 40 mmHg
- > 45 mmHg
- > 50 mmHg
- > 55 mmHg
- > 60 mmHg

1. Polytrauma patient

- > 40 mmHg
- > 45 mmHg
- > 50 mmHg
- > 55 mmHg
- > 60 mmHg

1. Resuscitated patient

- > 40 mmHg
- > 45 mmHg
- > 50 mmHg
- > 55 mmHg
- > 60 mmHg

1. Septic patient

- > 40 mmHg
- > 45 mmHg
- > 50 mmHg
- > 55 mmHg
- > 60 mmHg

1. Other: ___________

**Based on the patient's disease severity, how important is it to you that...**

1. ...several alarm profiles (e.g., for COPD) are available?

- Strongly disagree
- Disagree
- Undecided
- Agree
- Strongly agree

1. …alarms sound louder or softer?

- Strongly disagree
- Disagree
- Undecided
- Agree
- Strongly agree

1. ...clinical decision support suggests alarm profiles based on artificial intelligence?

- Strongly disagree
- Disagree
- Undecided
- Agree
- Strongly agree

1. Other: ___________

**What advantages do you see when artificial intelligence creates patient-specific alarm profiles (e.g., for COPD) and continuously re-evaluates them?**

1. Less workload

- Strongly disagree
- Disagree
- Undecided
- Agree
- Strongly agree

1. Fewer false alarms

- Strongly disagree
- Disagree
- Undecided
- Agree
- Strongly agree

1. Increased patient safety

- Strongly disagree
- Disagree
- Undecided
- Agree
- Strongly agree

1. Other: ___________

**What dangers do you see when an artificial intelligence creates patient-specific alarm profiles (e.g., for COPD) and continuously re-evaluates them?**

1. Staff trust artificial intelligence too much

- Strongly disagree
- Disagree
- Undecided
- Agree
- Strongly agree

1. Staff loses control over alarm management

- Strongly disagree
- Disagree
- Undecided
- Agree
- Strongly agree

1. Staff loses clinical skills

- Strongly disagree
- Disagree
- Undecided
- Agree
- Strongly agree

1. Incorrect calculations by artificial intelligence due to incomplete patient record

- Strongly disagree
- Disagree
- Undecided
- Agree
- Strongly agree

1. Lack of transparency of artificial Intelligence calculations

- Strongly disagree
- Disagree
- Undecided
- Agree
- Strongly agree

1. Other: ___________

**Questions about technology affinity:**

1. I like to occupy myself in greater detail with technical systems.

- Completely disagree
- Largely disagree
- Slightly disagree
- Slightly agree
- Largely disagree
- Completely agree

1. I like testing the functions of new technical systems.

- Completely disagree
- Largely disagree
- Slightly disagree
- Slightly agree
- Largely disagree
- Completely agree

1. It is enough for me that a technical system works; I don’t care how or why.

- Completely disagree
- Largely disagree
- Slightly disagree
- Slightly agree
- Largely disagree
- Completely agree

1. It is enough for me to know the basic functions of a technical system.

- Completely disagree
- Largely disagree
- Slightly disagree
- Slightly agree
- Largely disagree
- Completely agree

1. Which age category do you belong to?

- 18-24 years
- 25-34 years
- 35-44 years
- 45-54 years
- 55-64 years
- > 65 years
