# Supplementary material for: Staff perspectives on the influence of patient characteristics on alarm management in the intensive care unit: a cross-sectional survey study
Source: BMC Health Serv Res. 2023 Jul 5;23:729. doi: 10.1186/s12913-023-09688-x (PMC10324165; doi:10.1186/s12913-023-09688-x)
Supplement: Supplementary file 4 — Supplementary Material 4 [file 12913_2023_9688_MOESM4_ESM.pdf]

ICMJE DISCLOSURE FORM

**Date:**	**8/8/2022**
**Your Name:**	**Univ.-Prof.Dr.med. Claudia Spies,ML**
**Manuscript Title:**	**The Influence of Patient Characteristics on Alarm Management in the Intensive Care Unit: Cross-sectional Survey Study**
**Manuscript Number (if known):**	Click or tap here to enter text.

In the interest of transparency, we ask you to disclose all relationships/activities/interests listed below that are related to the content of your manuscript. "Related" means any relation with for-profit or not-for-profit third parties whose interests may be affected by the content of the manuscript. Disclosure represents a commitment to transparency and does not necessarily indicate a bias. If you are in doubt about whether to list a relationship/activity/interest, it is preferable that you do so.

The author's relationships/activities/interests should be defined broadly. For example, if your manuscript pertains to the epidemiology of hypertension, you should declare all relationships with manufacturers of antihypertensive medication, even if that medication is not mentioned in the manuscript.

In item #1 below, report all support for the work reported in this manuscript without time limit. For all other items, the time frame for disclosure is the past 36 months.

		Name all entities with whom you have this relationship or indicate none (add rows as needed)	Specifications/Comments (e.g., if payments were made to you or to your institution)
		**Time frame: Since the initial planning of the work**	
1	All support for the present manuscript (e.g., funding, provision of study materials, medical writing, article processing charges, etc.) **No time limit for this item.**	☒ **None**	
		**Time frame: past 36 months**	
2	Grants or contracts from any entity (if not indicated in item #1 above).	☐ **None**	
		Deutsche Forschungsgemeinschaft / German Research Society	Public / reviewed grant
		Deutsches Zentrum für Luft- und Raumfahrt e. V. (DLR) / German Aerospace Center	Public / reviewed grant
		Einstein Stiftung Berlin/ Einstein Foundation Berlin	Public / reviewed grant
		Gemeinsamer Bundesausschuss / Federal Joint Committee (G-BA)	Public / reviewed grant
		Inneruniversitäre Forschungsförderung / Inner University Grants	Public / reviewed grant
		Projektträger im DLR / Project Management Agency	Public / reviewed grant
		Stifterverband/ Non-Profit Society Promoting Science and Education	Public / reviewed grant
		European Society of Anaesthesiology and Intensive Care	Public / reviewed grant
		BMWI - Federal Ministry for Economic Affairs and Climate Action	ZIM, KF2027803KJ2
		Baxter Deutschland GmbH	Industry 2020: Sponsorship for Leopoldina-Symposium
		Cytosorbents Europe GmbH	Industry 2020: Sponsorship for Leopoldina-Symposium
		Edwards Lifesciences Germany GmbH	Industry

		Name all entities with whom you have this relationship or indicate none (add rows as needed)	Specifications/Comments (e.g., if payments were made to you or to your institution)
			2020: Sponsorship for Leopoldina-Symposium
		Fresenius Medical Care	Industry<br>2020: Sponsorship for Leopoldina-Symposium
		Grünenthal GmbH	Industry<br>2020: Sponsorship for Leopoldina-Symposium
		Masimo Europe Ltd.	Industry<br>2017&2020: Sponsorship for Leopoldina-Symposium
		Pfizer Pharma PFE GmbH	Industry<br>2020: Sponsorship for Leopoldina-Symposium, Sponsorship for "Infektiologisches Symposium"
		Georg Thieme Verlag	Payment for book contribution as author
		Dr. F. Köhler Chemie GmbH	IIT; CESARO Study, Phydelio Study, LoveMi Study
		Sintetica GmbH	PODSPA Study & Industry<br>2020: Sponsorship for Leopoldina-Symposium
		Stifterverband für die deutsche Wissenschaft e.V. / Philips	Non pharmalogical delirium prevention shared &decision allocation
		Stiftung Charité	CSC Sepsis-Center
		AGUETTANT Deutschland GmbH	Industry<br>2020: Sponsorship for Leopoldina-Symposium
		AbbVie Deutschland GmbH & Co. KG	Industry<br>2020: Sponsorship for Leopoldina-Symposium
		Amomed Pharma GmbH	Industry<br>2020: Sponsorship for Leopoldina-Symposium
		InTouch Health	Industry<br>2020: Sponsorship for Leopoldina-Symposium
		Copra System GmbH	Industry<br>2020: Sponsorship for Leopoldina-Symposium
		Correvio GmbH	Industry<br>2020: Sponsorship for Leopoldina-Symposium
		Drägerwerk AG & Co. KGaA	IIT, not reviewed Industry<br>Ongoing -Industry 2020 Sponsorship for Leopoldina-Symposium
		Gemeinsamer Bundesausschuss / Federal Joint Committee (G-BA) -Innovationsfond	2019-2023<br>PRÄP-GO
		Max-Planck-Gesellschaft zur Förderung der Wissenschaften e.V.	ERONA 2019-2021
		Deutsche Gesellschaft für Anästhesiologie & Intensivmedizin (DGAI	DissoLVE 2019-2020
		Stifterverband für die deutsche Wissenschaft e.V. / Metronic	E-Health shared decision allocation (2018-2024)
		Philips ElectronicsNederland BV	2019 - 2020: ICU Feel Better App
		BMBF / RKI	2020-2022: TICO-COVID-19;<br>FUTURE-MOCCA; FUTURE UGA; FUTURE UZB; FUTURE ZAF
		BMBF - Federal Ministry of Education and Research	2020: CEO-sys ; EViPan Unimed
		Deutsche Forschungsgemeinschaft / German Research Society	2020: "Heisenberg professorship"
		Gemeinsamer Bundesausschuss / Federal Joint Committee (G-BA) -Innovationsfond	2020 SEPWISS
		BMBF	2021: STAIRS
		Gemeinsamer Bundesausschuss / Federal Joint Committee (G-BA) –Innovationsfond	2022 iWILL
		Gemeinsamer Bundesausschuss / Federal Joint Committee (G-BA) -Innovationsfond	2022 E=MC²
		Gemeinsamer Bundesausschuss / Federal Joint Committee (G-BA) –Innovationsfond	2022 DigiPOD
3	Royalties or licenses	☒ **None**	
4	Consulting fees	☒ **None**	
5	Payment or honoraria for lectures, presentations, speakers ureaus, manuscript writing or educational events	☒ **None**	

		Name all entities with whom you have this relationship or indicate none (add rows as needed)	Specifications/Comments (e.g., if payments were made to you or to your institution)
6	Payment for expert testimony	☒ None	
7	Support for attending meetings and/or travel	☒ None	
8	Patents planned, issued or pending	☐ None	
		15753 627.7	Issued EUROPE (GER;AT;CH;LI;DE;FR;GB;NL) **Inventor**
		PCT/EP 2015/067731	Issued US Patent; **Inventor**
		3 174 588	Issued EUROPE (GER;CH;LI;DE;FR;NL) **Inventor**
		10 2014 215 211.9	International Patent Germany
		10 2018 114 364.8	International Patent Germany
		10 2018 110 275.5	International Patent Germany
		50 2015 010 534.8	International Patent Germany
		50 2015 010 347.7	International Patent Germany
		10 2014 215 212.7	International Patent Germany
9	Participation on a Data Safety Monitoring Board or Advisory Board	☐ None	
10	Leadership or fiduciary role in other board, society, committee or advocacy group, paid or unpaid	☐ None	
		AWMF (Association of the Scientific Medical Societies in Germany)	unpaid
		Deutsche Forschungsgemeinschaft (German Research Foundation) review boards	unpaid
		Deutsche Akademie der Naturforscher Leopoldina e. V. – German National Academy of Sciences – Leopoldina	unpaid
11	Stock or stock options	☒ None	
12	Receipt of equipment, materials, drugs, medical writing, gifts or other services	☒ None	
13	Other financial or non-financial interests	☒ None	

Please place an "X" next to the following statement to indicate your agreement:

☒ I certify that I have answered every question and have not altered the wording of any of the questions on this form.

10/08/21
